# Maternal Death From Postpartum Necrotizing Fasciitis Arising in an Episiotomy: A Case Report

**DOI:** 10.1155/S1064744997000598

**Published:** 1997

**Authors:** Catherine M. Lynch, Donna M. Pinelli, C. Wayne Cruse, William N. Spellacy, John T. Sinnott, Ron G. Shashy

**Affiliations:** 1 Department of Obstetrics and Gynecology University of South Florida 4 Columbia Drive Suite 529 Tampa FL 33606 USA; 2 Department of Surgery Division of Aesthetic and Reconstructive Surgery University of South Florida Tampa FL USA; 3 Department of Medicine Division of Infectious Diseases University of South Florida Tampa FL USA

## Abstract

*Background:* Necrotizing fasciitis is a rare condition. We report a fatal case arising from an episiotomy in a previously healthy woman.

*Case:* A healthy 23-year-old prima gravida white female underwent vaginal delivery with repair of a proctoepisiotomy. Eighty-four hours postpartum, she developed increasing perineal swelling with severe pain. She presented on the 4th postpartum day with edema, erythema localized to the perineum, and vital signs significant only for tachycardia of 120/min. With a leukocytosis of 45,000/μl (87%) neutrophils, she was admitted to the hospital with an initial diagnosis of perineal cellulitis and empirically started on broad-spectrum intravenous antibiotic therapy. The patient's condition continued to deteriorate and she was then transferred to our facility on postpartum day 9 where a team performed two radical debridements of all necrotic tissue. Despite this and a broadened antibiotic coverage, the patient eventually experienced cardiopulmonary arrest and died on postpartum day 12.

*Conclusion:* Necrotizing fasciitis must be considered in the differential diagnosis of the postpartum patient presenting with severe vulvar pain and erythema. Our patient exemplifies the obscure presentation with seemingly minimal skin changes. Any delay in diagnosis and treatment, which must include expeditious aggressive surgical debridement, will likely result in severe morbidity or mortality.


Infectious Diseases in Obstetrics and Gynecology 5:341–344 (1997)
© 1998 Wiley-Liss, Inc.

### Maternal Death From Postpartum Necrotizing Fasciitis Arising in an Episiotomy: A Case Report

**Catherine M. Lynch,[1]* Donna M. Pinelli,[1] C. Wayne Cruse,[2] William N. Spellacy,[1] John T. Sinnott,[3] and Ron G. Shashy[1]**

*[1]Department of Obstetrics and Gynecology, University of South Florida, Tampa, FL*
*[2]Department of Surgery, Division of Aesthetic and Reconstructive Surgery, University of South Florida, Tampa, FL*
*[3]Department of Medicine, Division of Infectious Diseases, University of South Florida, Tampa, FL*

### Abstract

***Background:*** **Necrotizing fasciitis is a rare condition. We report a fatal case arising from an episiotomy in a previously healthy woman.**

***Case:*** **A healthy 23-year-old prima gravida white female underwent vaginal delivery with repair of a proctoepisiotomy. Eighty-four hours postpartum, she developed increasing perineal swelling with severe pain. She presented on the 4th postpartum day with edema, erythema localized to the perineum, and vital signs significant only for tachycardia of 120/min. With a leukocytosis of 45,000/µl (87%) neutrophils, she was admitted to the hospital with an initial diagnosis of perineal cellulitis and empirically started on broad-spectrum intravenous antibiotic therapy. The patient's condition continued to deteriorate and she was then transferred to our facility on postpartum day 9 where a team performed two radical debridements of all necrotic tissue. Despite this and a broadened antibiotic coverage, the patient eventually experienced cardiopulmonary arrest and died on postpartum day 12.**

***Conclusion:*** **Necrotizing fasciitis must be considered in the differential diagnosis of the postpartum patient presenting with severe vulvar pain and erythema. Our patient exemplifies the obscure presentation with seemingly minimal skin changes. Any delay in diagnosis and treatment, which must include expeditious aggressive surgical debridement, will likely result in severe morbidity or mortality. Infect. Dis. Obstet. Gynecol. 5:341–344, 1997.** © 1998 Wiley-Liss, Inc.

Key words infection; surgical debridement; postpartum necrotizing fasciitis; proctoepisiotomy

In the past 20 years there have been 8 case reports in the world literature regarding necrotizing fasciitis in the postpartum period.[1,7,8] We report a fatal case of necrotizing fasciitis arising in an episiotomy site in a previously healthy woman.

### CASE REPORT

A healthy-23-year-old prima gravida white female with an uncomplicated prenatal course underwent a vacuum-assisted vaginal delivery with repair of a fourth degree episiotomy (proctoepisotomy). No antibiotics were administered for this tear. She was discharged with a healthy male infant 36 h later with no postpartum complications. Over the next 48 h the patient developed increasing perineal swelling. Severe pain and malaise ensued, making her unable to care for her infant.

Due to her worsening condition, she presented to a local hospital on the 4th postpartum day. The involved area was edematous and erythematous and appeared to localize to the perineum. Vital signs were blood pressure 118/72 mmHg, respira-

*Correspondence to: Dr. Catherine Lynch, Department of Obstetrics and Gynecology, University of South Florida, 4 Columbia Drive, Suite 529, Tampa, FL 33606.

Received 23 June 1997

Accepted 3 November 1997

tions 20/min, pulse 120/min, and temperature 97.5°F. Significant laboratory studies included a white blood cell count of 45,000/μl (87% neutrophils), hemoglobin 15.6 g/dl, platelets 194,000/μl, $CO_2$ of 22 mmol/l, glucose 126 mg/dl, creatinine 1.1 mg/dl, and albumin 2.2 g/dl. She was admitted with an initial diagnosis of perineal cellulitis at the episiotomy site.

Broad-spectrum antibiotics (ampicillin, gentamicin, clindamycin) were empirically initiated in addition to local wound care (sitz baths and betadine). No debridement was undertaken. Despite this, the patient complained of increasing pelvic and left leg pain and inability to ambulate. As her pain was felt to be out of proportion to her physical findings, a physical therapy consult was obtained to aid in ambulation. Over the next 2 hospital days, the erythema and induration progressed, extending from the perineum to the inner thigh and to the buttock region. On postpartum day 6, wound and vaginal cultures returned, identifying growth of *Streptococcus bovis* and *Enterococcus*. Based on sensitivity results, antibiotic treatment was altered to replace ampicillin with ticarcillin/clavulanic acid.

On the 8th postpartum day, the patient displayed increasing anesthesia over the perineum, with extensive swelling and induration. The patient was still unable to ambulate despite physical therapy consultation. She remained afebrile but her white blood cell count had risen to 50,000/μl (74% neutrophils). Other significant laboratory findings were as follows: lactate dehydrogenase 206 U/l (94–172 U/l), creatinine kinase 537 mg/dl (38–173 mg/dl), SGOT 54 U/l (12–48 U/l), and calcium 6.8 mg/dl (8.7–10.7 mg/dl). At this time, the patient was referred to our facility for further care.

At the time of admission (postpartum day 9), a diagnosis of necrotizing fasciitis was made. This large area was discolored (purple to red), nontender, and indurated (Fig. 1). There was marked vulvar edema with bullae, crepitus, and paresthesia. It involved the perineum and extended bilaterally superiorly to just below the axilla and inferiorly to just above the knees. Vital signs were blood pressure 123/75 mmHg, respirations 14/min, pulse 110/min, and temperature 99.7°F. Significant laboratory findings were a white blood cell count of 55,000/μl (91% neutrophils), hemoglobin 13.4 g/dl, platelets 229,000/μl, $CO_2$ of 22 mmol/l, glucose 133 mg/dl, and creatinine 0.7 mg/dl. Intravenous penicillin G, gentamicin, and clindamycin were administered at maximal dosages. Five hours after transfer, a combined plastic surgery and gynecology team performed a radical debridement of all necrotic tissue of the abdomen, perineum, and thighs (Fig. 2).

An intraoperative infectious disease consult was obtained and aggressive intensive care was continued postoperatively (postpartum day 10). The antibiotic coverage was broadened with piperacillin/tazobactam, vancomycin, and gentamicin at the recommendation of the infectious disease consultant.

Despite this, the patient deteriorated. Her blood urine and wound cultures drawn after hospital transfer continued to be negative. She was intubated and required fluid support and blood products. Thirty-six units of packed red cells and 9 units of fresh frozen plasma were administered over the next 24 h. A second debridement was performed on the 11th postpartum day with removal of minimal tissue. The patient continued to experience massive fluid losses from the open debridement sites. On the 12th postpartum day, the patient's situation worsened and she eventually experienced cardiopulmonary arrest. Resuscitation attempts failed and the patient died 48 h after hospital transfer.

Postmortem examination revealed necrotizing fasciitis and necrotizing myositis. Status post extensive debridement of the perineum, lower abdominal wall, and left inner upper thigh, the surgical wounds were grossly free of necrosis and infection. There was diffuse alveolar damage present with pulmonary microthrombi consistent with sepsis with acute respiratory distress syndrome (ARDS), and an incidental finding of a serous cystadenoma of the left ovary.

### DISCUSSION

Necrotizing fasciitis was first reported as "hospital gangrene" in 1871 by confederate army surgeon Joseph Jones, M.D.[2] It was subsequently further described by Meleney in 1924 and is therefore also known as Meleney's gangrene.[3] Meleney was the first to describe the necessity for early surgical intervention in the treatment of this disease.[4] Necrotizing fasciitis is often associated with patients who are immunocompromised, diabetic, or have other predisposing conditions including trauma,

Fig. 1. Marked vulvar edema with early tissue necrosis on medial aspect of the left thigh. Some bronze discoloration is noted involving the left perineum.

Fig. 2. Debrided wound. The operator's fingers are within the rectum demonstrating the original proctoepisiotomy.

extensive surgery, radiation therapy, or chemotherapy.[5,6]

Obstetrical trauma was the only risk factor for necrotizing fasciitis in our patient. Necrotizing fasciitis is typically described as deep pain out of proportion to clinical findings associated with superficial paresthesia over the involved area. Pathophysiologically, this entity is believed to first irritate and then destroy nerve fibers. Early clinical signs include local edema, induration, skin discoloration, and hypoaesthesia. As with our patient, superficial skin changes may be minimal and thus obscure the diagnosis. Despite this innocuous appearance, the disease constantly progresses to involve deeper tissue levels, specifically to the level of the fascia. Fascial involvement with nerve compromise results in rapid progression of analgesia.

In general, this syndrome is caused by a polymicrobial infection which may or may not include *Clostridium*. Organisms that have been associated with necrotizing fasciitis following a vaginal delivery include *Streptococcus, Escherichia coli, Klebsiella, Pseudomonas aeruginosa, Proteus, Bacteroides fragilis, Peptostreptococcus, Peptococcus,* group A beta hemolytic *Streptococcus,* group B beta hemolytic *Streptococcus,* and *Staphylococcus aureus.*[1,7–9]

Early aggressive surgical debridement is the mainstay of therapy for this disease. Stephenson et al.[9] reviewed the records of 29 non-pregnant patients with necrotizing fasciitis of the vulva and found that a delay in diagnosis of >48 h resulted in 73% mortality. In our case, there was a 5-day delay before surgery was performed at the tertiary care center. Once surgical debridement is performed on these patients, the postoperative management is often complicated by hypoalbuminemia with associated low oncotic pressure and increased capillary permeability. These patients should be managed by surgeons and critical care teams experienced in the care of the critically ill patient.[10,11]

Necrotizing fasciitis is a rare complication in vaginal delivery. It must be considered in the differential diagnosis of the postpartum patient presenting with severe vulvar pain and erythema. Our patient exemplifies the obscure presentation of the disease with seemingly minimal skin changes. This differential is paramount especially when the patient presents with pain out of proportion to the clinical findings. Once necrotizing fasciitis is entertained as a potential diagnosis, immediate and aggressive diagnostic therapy must be undertaken. The first line therapy is aggressive surgical debridement with concomitant broad-spectrum intravenous antibiotic therapy. As illustrated by our case, any delay in diagnosis and delivering treatment will likely result in severe morbidity or mortality.[9]

### REFERENCES

1. Sutton GP, Smirz LR, Clark DH, Bennett JE: Group B streptococcal necrotizing fasciitis arising from an episiotomy. Obstet Gynecol 66:733–736, 1985.
2. Schneider GT: A postpartum emergency—Necrotizing fasciitis. Contemp OB/GYN 109–114, February 1988.
3. Meleny FL: A Treatise on Surgical Disease. Oxford: Oxford University Press, p 12, 1948.
4. Nolan TE, King LA, Smith EP, Gallup DC: Necrotizing surgical infection and necrotizing fasciitis in obstetric and gynecologic patients. South Med J 86:1363–1367, 1993.
5. Hoffman MS, Turnquest D: Necrotizing fasciitis of the vulva during chemotherapy. Obstet Gynecol 74:483–484, 1989.
6. Addison WA, Livengood CH, Hill GB, Sutton GP, Fortier KJ: Necrotizing fasciitis of vulvar origin in diabetic patients. Obstet Gynecol 63:473–478, 1984.
7. Ammari NN, Hasweh YG, Hassan AA, Karyoute S: Postpartum necrotizing fasciitis. Case report. Br J Obstet Gynaecol 93:82–83, 1986.
8. Piper JM, West P: Necrotizing fasciitis following postpartum tubal ligation. A case report and review of the literature. Arch Gynecol Obstet 256:35–38, 1995.
9. Stephensen H, Dotters DJ, Katz V, Droegemueller W: Necrotizing fasciitis of the vulva. Am J Obstet Gynecol 166:1324–1327, 1992.
10. Stamenkovic I, Lew PD: Early recognition of potentially fatal necrotizing fasciitis. The use of frozen-section biopsy. N Engl J Med 310:1689–1693, 1984.
11. Salvino C, Harford FJ, Dobrin PB: Necrotizing infections of the perineum. South Med J 86:908–911, 1993.
